# Association between Human Papillomavirus and Human T-Lymphotropic Virus in Indigenous Women from the Peruvian Amazon

**DOI:** 10.1371/journal.pone.0044240

**Published:** 2012-08-29

**Authors:** Magaly M. Blas, Isaac E. Alva, Patricia J. Garcia, Cesar Carcamo, Silvia M. Montano, Ricardo Muñante, Joseph R. Zunt

**Affiliations:** 1 Epidemiology, STD and HIV Unit, School of Public Health and Administration, Universidad Peruana Cayetano Heredia, Lima, Peru; 2 US Naval Medical Research Unit No. 6 (NAMRU-6), Lima, Peru; 3 Ucayali Regional Health Directorate, Ministry of Health, Ucayali, Peru; 4 Department of Epidemiology, School of Public Health, University of Washington, Seattle, Washington, United States of America; 5 Department of Neurology, Global Health and Medicine, School of Medicine, University of Washington, Seattle, Washington, United States of America; Asociacion Civil Impacta Salud y Educacion, Peru

## Abstract

**Background:**

No association between the Human T-cell lymphotropic virus (HTLV), an oncogenic virus that alters host immunity, and the Human Papillomavirus (HPV) has previously been reported. Examining the association between these two viruses may permit the identification of a population at increased risk for developing cervical cancer.

**Methods and Findings:**

Between July 2010 and February 2011, we conducted a cross-sectional study among indigenous Amazonian Peruvian women from the Shipibo-Konibo ethnic group, a group with endemic HTLV infection. We recruited women between 15 and 39 years of age who were living in the cities of Lima and Ucayali. Our objectives were to determine the association between HTLV and: (i) HPV infection of any type, and (ii) high-risk HPV type infection. Sexually active Shipibo-Konibo women were screened for HTLV-1 and HTLV-2 infections. All HTLV-1 or -2 positive women, along with two community-matched HTLV negative sexually active Shipibo-Konibo controls were later tested for the presence of HPV DNA, conventional cytology, and HIV. We screened 1,253 Shipibo-Konibo women, observing a prevalence of 5.9% (n = 74) for HTLV-1 and 3.8% (n = 47) for HTLV-2 infections. We enrolled 62 (60.8%) HTLV-1 positive women, 40 (39.2%) HTLV-2 positive women, and 205 community-matched HTLV negative controls. HTLV-1 infection was strongly associated with HPV infection of any type (43.6% vs. 29.3%; Prevalence Ratio (PR): 2.10, 95% CI: 1.53–2.87), and with high-risk HPV infection (32.3% vs. 22.4%; PR: 1.93, 95% CI: 1.04–3.59). HTLV-2 was not significantly associated with either of these HPV infections.

**Conclusions:**

HTLV-1 infection was associated with HPV infection of any type and with high-risk HPV infection. Future longitudinal studies are needed to evaluate the incidence of high-risk HPV infection as well as the incidence of cervical neoplasia among HTLV-1 positive women.

## Introduction

Human viruses are associated with approximately 10–15% of human malignancies worldwide or approximately 1.3 million new cancer cases each year [1]. Cervical cancer is the second most common cancer in women worldwide and the leading cause of cancer-related deaths among women in Peru [2]–[4]. The cause of cervical cancer is infection by human papillomavirus (HPV), a virus transmitted primarily through sexual intercourse [5]–[7]. HPV prevalence in Peru is 7.5% among women with normal cytology [4].

Certain HPV genotypes are oncogenic. These high-risk types are associated with the development of cervical cancer and with cervical intraepithelial neoplasia (CIN) stage 3, a precursor to invasive cervical cancer [8]. In general, impaired host immunity (such as the one associated with HIV infection) allows the persistence of high-risk HPV viruses and thus increases the risk for developing CIN [9]–[13].

HIV and Human T-lymphotropic virus (HTLV) are both oncogenic retroviruses that target CD4+ T cells, which play a role in controlling HPV infection [14], [15]. HIV is correlated with progression from asymptomatic HPV infection to cervical cancer [10]–[12]. Whether this correlation also occurs with HTLV is unknown. The potential association between HTLV and HPV infections has likely received little attention because HTLV infection is endemic in geographic areas that are located mostly in resource-constrained settings [15].

HTLV infects 10 to 20 million people worldwide and is endemic in southern Japan, the Caribbean, the Melanesian islands, Papua New Guinea, the Middle East, Central and South Africa and South America [16]–[18]. All 13 South American countries have reported the presence of HTLV infection. The highest HTLV prevalences in the general population have been reported in Brazil, Colombia, and Peru [19]. A 2007 study found a pocket of HTLV infection among the Shipibo-Konibo people, an Amazonian ethnic group from Peru [20].

To our knowledge, there have been no reports regarding the association between HPV and HTLV infections. Examining the association between these two viruses may permit the identification of a population at increased risk for developing cervical cancer. The objectives of this study were to determine the association between HTLV and: (i) any HPV infection, and (ii) high-risk HPV infection.

## Methods

### Ethics statement

Our proposal and this study were approved by the Institutional Review Board of the University of Washington in Seattle and the Universidad Peruana Cayetano Heredia and U.S. Naval Medical Research Unit No.6 (NAMRU-6) in Lima, Peru. All enrollees provided a written informed consent prior to their participation in our study.

### Study Design

Between July 2010 and February 2011, we conducted a cross-sectional study among women from Shipibo-Konibo communities between 15 and 39 years of age who lived in two communities in Lima, the capital of Peru, or in 22 Shipibo-Konibo indigenous Amazon Jungle communities located within four hours by car or boat from the city of Pucallpa. Sexually active Shipibo-Konibo women with HTLV-1 or HTLV-2 infection, along with two community-matched HTLV negative sexually active Shipibo-Konibo controls were later tested for the presence of HPV DNA, conventional cervical cytology and HIV infection. We defined women as belonging to the Shipibo-Konibo ethnic group if they had at least one of the following characteristics: 1) they self-identified as Shipibo-Konibo, 2) they spoke Shipibo-Konibo language, or 3) at least one of their parents belonged to the Shipibo-Konibo ethnic group.

### Visits to the communities

In our study, we first established community advisory boards (CABs) in Lima and in Ucayali. The objective of these CABs was to provide advice regarding approaches to engage the communities in the project and to receive feedback regarding study procedures. These CABs included leaders of the communities involved in the study, key personnel from the indigenous health program of the Interethnic Association for the Development of the Peruvian Rainforest (AIDESEP), and personnel from the Ministry of Health and Non Governmental Organizations working with indigenous peoples.

After receiving feedback from the CABs, we initiated visits to the communities. The study visits were divided into two phases. In the first phase, we visited the chief of each community selected for the project. If the chief agreed to permit the community to be involved in the study, he signed the informed consent; we then conducted a census of the community to identify women who met our inclusion criteria. Later, we individually approached all eligible women at their home to assess their interest in participation. If participants were at least 18 years old, or between 15 and 17 years of age and married/cohabitating for two or more years, and they agreed to participate, they were invited to participate and asked to sign the informed consent. Other consenting eligible women signed an assent and their parent or legal guardian signed an informed consent. Afterwards, we administered a questionnaire and took a venous blood sample to test for HTLV infection.

Approximately two months after the first visit, we carried out a second visit to the communities to deliver HTLV test results. During the second phase, we selected all women who tested positive for HTLV and for each HTLV-positive women, two randomly selected HTLV-negative women from the same community. After these participants signed a second informed consent, a midwife performed a speculum examination of the cervix, collected a cervical sample with a polyester swab, a sample with an Ayre spatula, and a cytobrush specimen for cytology. The polyester swabs were placed in a 4.5 ml cryotube containing 1 ml of Specimen Transport Medium (STM, Digene Corp., Gaithersburg, MD) and transported in a cooler to the laboratory where they were stored at −20° until shipment to the University of Washington. The Pap smears were fixed with 96% ethanol for at least 15 minutes and then placed in special boxes prior to their shipment to Lima. The stored blood samples from all participants selected for the second phase of the study were tested for HIV infection.

All women with abnormal cytologic results were referred for colposcopy and offered free treatment, when indicated. Women testing positive for HIV infection were referred to the Ministry of Health, where free highly active antiretroviral therapy is provided.

### Survey

During the first and second visits, we performed a survey to obtain demographic characteristics, sexual and other risk behaviors for Sexually Transmitted Infections (STIs), STI history, smoking status, drug and alcohol use, parity, abortion, contraceptive use, history of previous Pap smears, history of cervical abnormalities, knowledge about HPV and willingness to get vaccinated against this virus, risk factors for HTLV acquisition and history of HTLV related symptoms. The survey was administered in Spanish since in previous studies researchers identified that most women under 40 years living in that area spoke Spanish. We piloted the survey for understanding of language and accurate interpretation of questions prior to initiating the surveys.

### Data collection tool

Survey information was collected using EpiSurveyor Mobile [21], a mobile phone based application for collecting data in field-based surveys. With this application the interviewer was able to fill the survey with a point-and-click interface. Some elements of the survey included check boxes, numeric-entry boxes, and selection lists to facilitate data entry. Once the data was collected in the cell phone at the communities, study personnel returned to the city and downloaded the information via WiFi or through a phone-memory card directly to the database.

### Laboratory techniques

#### HTLV testing

HTLV testing was performed at NAMRU-6 in Peru. HTLV-1 and -2 antibody testing was conducted using ELISA (Vironostika, North Carolina) with confirmatory Western blot assay (HTLV-1/2 Blot 2.4, Genelabs Diagnostics, Singapore). A woman was considered HTLV-1 seropositive if the ELISA assay was positive and confirmatory Western blot assay revealed bands representing gag (p24, p19), gp46, and two env proteins (GD21 and rgp 46-I). An individual was considered HTLV-2 seropositive if the ELISA assay was positive and confirmatory Western blot assay revealed bands representing p24, GD21 and rgp46-II. If gag and env proteins were absent, but other HTLV specific bands, such as p53 or p19 were present, the individual was considered indeterminate. Subjects with indeterminate results were excluded from the analysis.

#### HPV testing

For HPV testing we used linear array assay (Roche Molecular Systems). For HPV DNA preparation, samples were digested with 20 μg/mL proteinase K for 1–2 h at 37°C. For each sample, 2400 μL was used to isolate DNA following the manufacturer's protocol (QIAamp DNA Blood Mini Kit, Qiagen Sciences, Gaithersburg, MD). Then, each DNA sample was amplified using HPV L1 consensus primers MY09, MY11, and HMB01 and β-globin primers PC04 and GH20. Five µl of PCR products were then dotted onto nylon filters and probed with biotin-labeled β-globin and HPV generic probes. Samples negative for β-globin were considered insufficient for HPV DNA testing.

Samples testing positive by the generic HPV probe were typed using the line-blot assay developed by Roche Diagnostics. This assay recognizes 37 individual HPV types: high-risk types, 16, 18, 26, 31, 33, 35, 39, 45, 51–53, 56, 58, 59, 66–68, 73, 82, and IS39; low-risk types, 6, 11, 40, 42, 54, 55, 61, 62, 64, 69–72, 81, 83, 84, and CP6108. For the analysis of high-risk HPV infection, we included types 16, 18, 31, 33, 35, 39, 45, 51, 52, 56, 58, 59, 66 as defined at the 2005 meeting of the International Agency for Research on Cancer [8].

#### Cytology

Cervical slides were sent to a cytopathologist in Lima for conventional cytology. Presence of endocervical or metaplastic cells were rated according to the Bethesda 2001 criteria [22].

#### HIV testing

We used a fourth-generation enzyme immunoassay (EIA) to detect HIV-1/2 IgG and IgM antibodies or HIV-1 p24 antigen (Genscreen ULTRA HIV Ag–Ab, Bio-Rad). Positive samples were confirmed by Western blot.

### Statistical Methods

A generalized linear model, matching by community of residence, was used to assess the association between HTLV and (i) any HPV infection, (ii) infection with high-risk HPV type, (iii) cytologic abnormalities in the cervix, and (iv) presence of a low-grade squamous intraepithelial lesion (LSIL) or higher lesion. Prevalence ratios (PR) estimates were adjusted for age, education, age of sexual partner, number of sexual partners within the last 12 months, and condom use at last sexual intercourse. Analyses were performed using Stata (v8.0; Stata Corp., College Station, TX).

## Results

Between August 2010 and February 2011, we identified 1,460 women, and 1,402 agreed to be screened for HTLV infection. Of these women, 149 were not Shipibo-Konibo women (13 were Ashaninka, three Awajun, one Cashinahua, one Isconahua, one Lamista, two Machiguenga, six Sharanahua, one Shawi, two Yanesha, 11 Yines, one Quechua andina, and 107 Mestizas) and were excluded from the analysis. Of the 1,253 eligible Shipibo-Konibo women, 74 (5.9%) tested positive for HTLV-1, 47 (3.8%) for HTLV-2 infection, and 4 (0.3%) had indeterminate results. Of the 121 HTLV-1 or -2 Shipibo-Konibo positive women, four were non-sexually active women; 11 migrated to other communities between the HTLV screening visit and the HPV testing visit; two did not agree to undergo the gynecologic examination, and two did not have controls from their community and were excluded from HPV testing. This analysis includes 62 (60.8%) HTLV-1 positive, 40 (39.2%) HTLV-2 positive, and 205 HTLV negative women who underwent HPV, Pap and HIV testing. Three of these women tested positive for HIV infection (two HIV positive women were negative for HTLV and one was positive for HTLV-2). These women were included in the analysis.

HTLV-infected women were more likely to be older and less educated than HTLV-negative women (Table 1). HTLV-infected women were also more likely than HTLV-negative participants to have a sexual partner either younger or of the same age and more likely to have not used condoms during last sexual intercourse.

**Table 1:** Demographic characteristics and sexual behaviors among women with and without HTLV Infection.

Variable	HTLV-1 or -2 Positive (n = 102)	HTLV Negative (n = 205)	PR (95% CI)	P
Age years (mean, SD)	28.3 (6.9)	26.0 (6.7)	1.03 (1.02–1.05)	**<0.001**
Able to read	98(96.1%)	198(96.6%)	0.91 (0.44–1.89)	0.80
Primary education or less	38 (37.3%)	50 (24.4%)	1.48 (1.01–2.16)	**0.04**
Married/Cohabitating	79(77.5%)	155(75.6%)	1.07 (0.82–1.40)	0.61
Age at first sexual intercourse (mean, SD)	14.8 (1.56)	15.1 (1.64)	0.99 (0.95–1.02)	0.50
Age at first pregnancy (mean, minimun-maximun)	16.8 (12–25)	17.3 (12–30)	0.96 (0.89–1.03)	0.28
Currently pregnant	16 (15.7%)	29 (14.2%)	1.08 (0.79–1.49)	0.62
Number of sexual partners within the last 12 months (mean, SD)	1.10 (0.70)	1.03 (0.48)	1.16 (0.88–1.53)	0.28
New sexual partner within the last 12 months	12 (11.9%)	20 (10.0%)	1.14 (0.67–1.93)	0.63
Received money for sexual intercourse	4(4.0%)	7 (3.5%)	1.09 (0.54–2.20)	0.81
Ever forced to have sexual intercourse	5(4.9%)	7(3.4%)	1.27 (0.76–2.12)	0.37
Ever had genital warts	6 (5.9%)	6 (2.9%)	1.54 (0.83–2.86)	0.18
Non-stable last sexual partner	8 (7.8%)	11 (5.4%)	1.29 (0.73–2.27)	0.38
Sexual partner younger or same age vs. older	30 (29.7%)	35 (17.2%)	1.55 (1.10–2.19)	**0.01**
Partner lives in another residence	22 (21.8%)	54 (26.7%)	0.83 (0.66–1.04)	0.11
Partner traveled within the last 12 months	49 (48.0%)	95 (46.8%)	1.03 (0.75–1.42)	0.84
Partner had an STI within the last 3 months	0(0)	6(3.0%)	*	0.18
Used condom at last sexual intercourse	3 (3.0%)	24 (11.8%)	0.31 (0.12–0.85)	**0.02**
Partner had another partner				
Yes	31(30.7%)	61 (30.1%)	1.00	
No	20 (19.8%)	46(22.7%)	0.90 (0.58–1.39)	0.64
Dońt know	50(49.5%)	96 (47.3%)	1.02 (0.72–1.43)	0.93
Parner belongs to the Shipibo-Konibo ethnic group	84 (84.0%)	169 (83.3%)	1.04 (0.76−1.41)	0.81
Participant worked at a camp lodge	6 (5.9%)	7 (3.4%)	1.41 (0.79−2.52)	0.24
Current smoker	5 (4.9%)	6 (2.9%)	1.39 (0.86–2.22)	0.18

* PR cannot be calculated because of empty cells. P values calculated using Fisher's exact test.

After adjustment for potential confounding variables, there was an association between HTLV-1 or -2 infection and the presence of any HPV type infection. There is the possibility of residual confounding for lifetime number of sexual partners, as we only adjusted for number of sexual partners within the last 12 months. We found a non-significant association between HTLV-1 or -2 infections and the presence of high-risk HPV infection, cervical cytologic abnormalities or presence of LSIL or higher lesion in the cervix (Table 2).

**Table 2:** Association between HTLV-1 or -2 infection and HPV infection and cytological abnormalities in women from the Shipibo-Konibo ethnic group.

	HTLV Negative (n = 205)	HTLV-1 or -2 Positive (n = 102)	HTLV-1 or -2 infection PR (95%CI)	
			Crude	Adjusted*
HPV infection with any type	60 (29.3%)	37 (36.3%)	1.23 (0.91–1.66)	1.42 (1.10–1.83)
High-risk HPV infection	46 (22.4%)	27 (26.5%)	1.15 (0.68–1.97)	1.35 (0.79–2.30)
Abnormal Papanicolaou †	24 (11.8%)	17 (16.8%)	1.30 (0.82–2.05)	1.29 (0.81–2.07)
LSIL or higher cytological abnormalities ‡	14 (6.9%)	9 (8.9%)	1.20 (0.67–2.14)	1.38 (0.73–2.58)

*Adjusted for age, education, age of sexual partner, number of sexual partners within the last 12 months, and condom use at last sexual intercourse.

† We included participants who had Atypical squamous cells of undetermined significance (ASC-US), Atypical squamous cells –01 cannot exclude HSIL (ASC-H), Atypical glandular cells of undetermined significance (AGUS), Low-grade squamous intraepithelial lesion (LSIL), High-grade squamous intraepithelial lesion (HSIL), Squamous cell carcinoma, and Adenocarcinoma in situ (AIS).

‡ We included participants who had LSIL, HSIL, Squamous cell carcinoma, and Adenocarcinoma in situ.

Women with HTLV-1 were two times more likely to have HPV infection of any type and high-risk HPV infection than HTLV negative women, after adjusting for confounding variables. However, HTLV-1 positive women had a similar prevalence of epithelial cell abnormalities in the cervix or presence of LSIL or higher grade lesion than women without HTLV (Table 3).

**Table 3:** Association between HTLV-1 infection and HPV infection and cytological abnormalities in women from the Shipibo-Konibo ethnic group.

	HTLV Negative (n0 = 205)	HTLV-1 Positive (n = 62)	HTLV-1 infection PR (95%CI)	
			Crude	Adjusted*
HPV infection with any type	60 (29.3%)	27 (43.6%)	1.60 (1.09–2.35)	2.10 (1.53–2.87)
High-risk HPV infection	46 (22.4%)	20 (32.3%)	1.45 (0.75–2.80)	1.93 (1.04–3.59)
Abnormal Papanicolaou †	24 (11.8%)	11 (18.0%)	1.44 (0.69–2.99)	1.39 (0.65–2.97)
LSIL or higher cytological abnormalities ‡	14 (6.9%)	7 (11.5%)	1.50 (0.68–3.33)	1.73 (0.68–4.44)

* Adjusted for age, education, age of sexual partner, number of sexual partners within the last 12 months, and condom use at last sexual intercourse.

† We included participants who had atypical squamous cells of undetermined significance (ASC-US), Atypical squamous cells - cannot exclude HSIL (ASC-H), Atypical glandular cells of undetermined significance (AGUS), Low-grade squamous intraepithelial lesion (LSIL), High-grade squamous intraepithelial lesion (HSIL), Squamous cell carcinoma, Adenocarcinoma in situ (AIS).

‡ We included participants who had LSIL, HSIL, Squamous cell carcinoma, and Adenocarcinoma in situ.

Women with HTLV-2 infection had a similar prevalence of HPV infection compared to HTLV seronegative women (25.0% vs. 29.3%, respectively; PR: 0.95, 95% CI: 0.55–1.64). They also had a similar prevalence of high-risk HPV type infection (18.0% vs. 22.4%; PR: 0.85, 95% CI: 0.36–2.00), abnormal Pap smear (15.0% vs. 11.8%; PR: 1.33, 95% CI: 0.64–2.79), or Pap smear with a LSIL lesion or higher lesion than controls (5.0% vs. 6.9%; PR: 0.90, 95% CI: 0.32–2.52).

**Figure 1:**
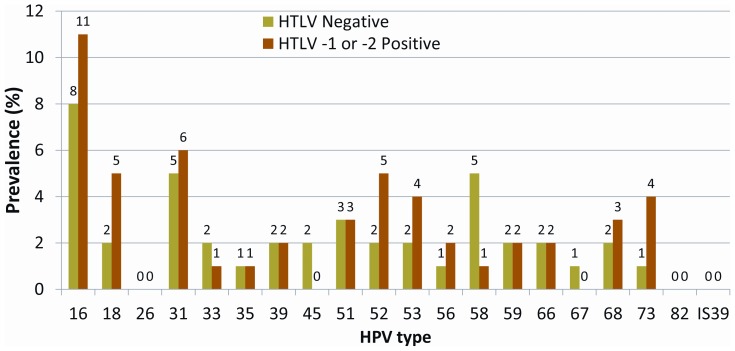
Prevalence of high-risk HPV among Shipibo-Konibo women with and without HTLV-1 or 2 infections.

Among women with HTLV-1 or -2 infection, the most frequent high-risk HPV type was HPV 16 (10.8%, n = 11), followed by HPV 31 (5.9%, n = 6), HPV 18 (4.9%, n = 5), and HPV 52 (4.9%, n = 5; Figure 1). The prevalence of HPV 6 was 3.9% (n = 4) and the prevalence of HPV 11 was 0%. The combined prevalence of HPV 16 or 18 infection was 15.7% (n = 16) and the combined prevalence of HPV 6, 11, 16 or 18 infection was 16.7% (n = 17).

Among women with HTLV-1 infection, the most frequent high-risk HPV type was HPV 16 (12.9%, n = 8), followed by HPV 18 (6.5%, n = 4), HPV 31 (6.5%, n = 4), HPV 52 (6.5%, n = 5), HPV 53 (6.5%, n = 5), and HPV 73 (6.5%, n = 4). The prevalence of HPV 6 was 4.8% (n = 3) and the prevalence of HPV 11 was 0%. The combined prevalence of HPV 16 or 18 infections was 19.4% (n = 12) and the combined prevalence of HPV 6, 11, 16 or 18 infection was 21.0% (n = 13).

## Discussion

To our knowledge, this is the first description of a potential association between HPV and HTLV infections. Women with HTLV-1 infection had increased prevalences of HPV infection of any type and with high-risk HPV infection. HPV prevalence in women with HTLV-2 infection was similar to that found in HTLV seronegative controls. Among HTLV-1 infected women, the prevalence of HPV infection with any type was 43.6% and the prevalence of high-risk HPV infection was 32.3%. These prevalences are higher than those reported in Peruvian women from the general population (7.5% for any HPV type and 12.6% for a high-risk HPV type) [4], [23] and surprisingly similar to rates in Peruvian female sex workers (50.6% of female sex workers had HPV infection of any type and 35.6% were infected with a high-risk HPV type) [24], [25].

The higher prevalence of HPV infection among HTLV-1 infected women could be due to a higher incidence of HPV infection among HTLV-1 seropositive women or to a longer duration of infection in these women. A higher incidence could also be the result of both viruses sharing sexual intercourse as a route of transmission; although HTLV-2 infection is also transmitted via sexual intercourse and was not similarly associated with higher HPV prevalence [15]. We could not adjust the prevalence ratio estimate for lifetime number of sexual partners, so the possibility of residual confounding remains. Compared to HTLV-2, HTLV-1 is more likely to produce clinical symptoms, such as HTLV-associated myelopathy and Adult T-cell leukemia/lymphoma via T lymphocyte dysfunction. As T cell function is important for controlling HPV infection, the relative T lymphocyte dysfunction in HTLV-1-infected women (none had clinically evident HTLV-1-associated disease) could modulate persistence of HPV infection [26].

We did not detect an association between HTLV infection and cervical cytologic abnormalities. It is important to mention that our study was not powered to detect this association. A case-control study conducted in Mexico, comparing women with and without cervical cancer also did not detect an association between HTLV infection and cervical cancer [27]. In contrast, a study conducted in Japan comparing women with cervical carcinoma from a hospital registry to age-matched healthy population-based controls found that HTLV-1 infection was associated with a nearly three times greater risk of cervical carcinoma in patients younger than 59 years and with a seven times greater risk of vaginal carcinoma in women of all ages [28].

If HTLV increases the risk for cervical cancer it is most likely through HPV infection. Two hospital-based case-control studies in Jamaica that assessed if HTLV infection increased the risk of cervical cancer above and beyond HPV infection did not find an association [29], [30]. In the study of Strickler et al., women with CIN-3 or invasive cancer (IC) were not more likely to have HTLV-1 infection than women with either CIN-1, koilocytotic atypia, atypical squamous cells of undetermined significance or benign cervical pathology [29]. In the study conducted by Castle et al., women with CIN-3 or cancer were not more likely to have HTLV-1 infection than women with CIN-1 [30].

HTLV has also been associated with extensive genital warts. Radja et al. reported two cases of severe condylomatosis associated with HTLV-1 infection in a Caribbean and Ghanaian women who lived in the UK [31]. In our study, we did not find an association between HTLV infection and self-report of genital warts. Another study reported an association between HTLV and cervicitis; in a study conducted by Zunt et al. on Peruvian female sex workers, HTLV tax DNA was associated with the presence of ≥30 polymorphonuclear cells within cervical mucus per 100 X microscopic field and with the presence of cervical secretions [32].

Our study has some potential limitations: 1) the cross sectional nature of this study precludes the determination of the temporal relationship between HTLV and HPV infection, and 2) the inclusion of prevalent cases of HPV infection means that any association with HTLV may be due to higher incidence or longer duration of infection.

In conclusion, HTLV-1 infection was associated with higher prevalence of any HPV type infection and with high-risk HPV type infection. Future studies to evaluate the persistence of high-risk HPV type infection as well as the incidence of cervical neoplasia among HTLV-1 positive women could examine the temporal relationship between HTLV and HPV infection and could have implications regarding whether HPV vaccination should be emphasized for HTLV-1 infected women.
